# Gender-Related Differences on Polyamine Metabolome in Liquid Biopsies by a Simple and Sensitive Two-Step Liquid-Liquid Extraction and LC-MS/MS

**DOI:** 10.3390/biom9120779

**Published:** 2019-11-26

**Authors:** Iris Samarra, Bruno Ramos-Molina, M Isabel Queipo-Ortuño, Francisco J Tinahones, Lluís Arola, Antoni Delpino-Rius, Pol Herrero, Núria Canela

**Affiliations:** 1 Eurecat, Centre Tecnològic de Catalunya, Centre for Omic Sciences (Joint Unit Eurecat-Universitat Rovira i Virgili), Unique Scientific and Technical Infrastructure (ICTS), 43204 Reus, Spain; iris.samarra@eurecat.org (I.S.); antoni.delpino@eurecat.org (A.D.-R.); pol.herrero@eurecat.org (P.H.); 2 Department of Endocrinology and Nutrition, Virgen de la Victoria University Hospital, Institute of Biomedical Research of Malaga (IBIMA), 29010 Malaga, Spain; brunoramosmolina@gmail.com (B.R.-M.); fjtinahones@hotmail.com (F.J.T.); 3 CIBER Physiopathology of Obesity and Nutrition (CIBERobn), Institute of Health Carlos III, 28029 Madrid, Spain; maribelqo@gmail.com; 4 Department of Medical Oncology, Virgen de la Victoria University Hospital, Institute of Biomedical Research of Malaga (IBIMA), 29010 Malaga, Spain; 5 Nutrigenomics Research Group, Biochemistry and Biotechnology Department, Universitat Rovira i Virgili, 43007 Tarragona, Spain; lluis.arola@urv.cat; 6 Eurecat, Centre Tecnològic de Catalunya, Biotechnological Area, 43204 Reus, Spain

**Keywords:** polyamines, acetylpolyamines, LC-MS/MS, obesity, polyamine metabolism, liquid biopsies

## Abstract

Polyamines are involved in the regulation of many cellular functions and are promising biomarkers of numerous physiological conditions. Since the concentrations of these compounds in biological fluids are low, sample extraction is one of the most critical steps of their analysis. Here, we developed a comprehensive, sensitive, robust, and high-throughput LC-MS/MS stable-isotope dilution method for the simultaneous determination of 19 metabolites related to polyamine metabolism, including polyamines, acetylated and diacetylated polyamines, precursors, and catabolites from liquid biopsies. The sample extraction was optimized to remove interfering compounds and to reduce matrix effects, thus being useful for large clinical studies. The method consists of two-step liquid-liquid extraction with a Folch extraction and ethyl acetate partitioning combined with dansyl chloride derivatization. The developed method was applied to a small gender-related trial concerning human serum and urine samples from 40 obese subjects. Sex differences were found for cadaverine, putrescine, 1,3-diaminopropane, γ-aminobutyric acid, N8-acetylspermidine, and N-acetylcadaverine in urine; N1-acetylspermine in serum; and spermine in both serum and urine. The results demonstrate that the developed method can be used to analyze biological samples for the study of polyamine metabolism and its association with human diseases.

## 1. Introduction

Polyamines are aliphatic amines with low molecular weights that are involved in the regulation of many cellular functions, including translation, transcription, signal transduction, cell proliferation, cell differentiation, apoptosis, and cell stress responses [1]. Polyamine metabolism is frequently dysregulated in many human pathologies, such as cancer, metabolic disorders, and autoimmune diseases [2,3]. The major polyamines in mammalian cells are spermidine (SPD), spermine (SPM), and their precursor, diamine putrescine (PUT). They are present in a variety of biological matrices in both their natural and acetylated forms. In addition to polyamines and acetylpolyamines, the polyamine metabolome includes their precursor amino acids (i.e., arginine (ARG), ornithine (ORN), and lysine (LYS)) as well as the polyamine-related metabolites produced by nonmammalian organisms, such as cadaverine (CAD), agmatine (AGM), and 1,3-diaminopropane (1,3-DAP) [4]. A comprehensive study of the polyamine metabolism has previously shown to be a promising target to discover new and reliable biomarkers in medicine and nutrition. For instance, urinary N1, N12-diacetylspermine (N1,N12-DiAcSPM), has been proposed as a biomarker for the diagnosis and prognosis of different types of cancer [3,5,6,7,8], and the plasma levels of N-acetylputrescine (N-AcPUT) and 1,3-DAP have been proposed as biomarkers for the early diagnosis of lung cancer [9].

In mammalian cells, polyamines ionically interact with nucleic acids, proteins, and other negatively charged molecules [10]. It has also been confirmed that the covalent binding of polyamines to proteins, catalyzed by transglutaminase, takes place in various mammalian tissues [11]. In this sense, free polyamines can be defined as the extractable polyamines without a hydrolysis step. Nowadays, most of the current research focused on biomarker discovery in biological samples pays more attention to free polyamines and related metabolites [12]. Various analytical methods have been developed for the determination of polyamines in liquid biopsies, such as serum or urine samples. Some authors have used gas chromatography-mass spectrometry (GC-MS) [13,14], enzyme-linked immunosorbent assays (ELISAs) [15], and liquid chromatography with fluorescence detection (LC-FLD) [16,17] among other methods, but the preferred method for polyamine analysis is liquid chromatography-mass spectrometry (LC-MS). Focusing on the LC-MS methods, the direct determination of polyamines is difficult due to their small size and aliphatic structure; however, it has been achieved mainly with the use of ion-pairing reagents, such as heptafluorobutyric acid [4,18,19,20,21] or perfluoroheptanoic acid [12]. The use of ion-pairing reagents is not advisable due to potential problems with the analysis in the LC-MS system since it is well known that these reagents cause ion source contamination and signal suppression when using electrospray ionization; this is in addition to causing a reduction in the analytical column performance [22]. Another widely used option is the use of a derivative agent to increase the hydrophobicity and ionization efficiency of polyamines. Some of the most common derivatives are dansyl chloride, benzoyl chloride [23,24,25], isobutyl chloroformate [26,27], N-(9-fluorenylmethoxycarbonyloxy)succinimide (FMOC) [28], 4-(N,N-dimethylaminosulfonyl)-7-fluoro-2,1,3-benzoxadiazole (DBD-F) [29,30], and 1-(5-fluoro-2,4-dinitrophenyl)-4-methylpiperazine (PPZ) [31]. Dansyl chloride derivatization has been extensively used for the analysis of polyamines and other biogenic amines in a variety of biological matrices, including plasma [32,33,34], red blood cells [35,36], and urine [37].

Since the circulating levels of polyamines are much lower than the intracellular concentration levels [10,38], sample extraction is one of the most critical steps when plasma or serum samples are analyzed. An optimized sample extraction procedure is required to maximize the performance of the method. One of the most common sample pretreatment methods on biofluids for the analysis of polyamines is protein precipitation with perchloric acid (PCA). This is usually followed by derivatization and liquid-liquid extraction (LLE) with an organic solvent, such as diethyl ether or dichloromethane [23,25,35,36]. As described by Magnes et al. [27], trichloroacetic acid can also be used as a protein precipitation agent followed by derivatization with isobutyl chloroformate. Another highly used option is the use of an organic solvent for protein precipitation, such as methanol (MeOH) or acetonitrile (ACN). MeOH is the preferred option when ion-pairing chromatography is used [4,12,18,19,21], but it has also been used with derivatizing agents [30,31]. ACN is also commonly used before derivatization with diverse reagents [24,28,29,32,33]. Cardeano et al. [34] proposed the use of ACN followed by dansyl chloride derivatization and LLE with ethyl acetate for the analysis of biogenic amines. Penthane has been used by Byun et al. [26] coupled with isobutyl chloroformate derivatization and an LLE with diethyl ether. Finally, solid phase extraction (SPE) is another sample pretreatment method used either with ion-pairing reagents [20] or derivatization [37,39,40].

Thus, the aim of the current study was to develop a comprehensive, sensitive, robust, and high-throughput LC-MS/MS method for the simultaneous determination of 19 free metabolites related to polyamine metabolism in human liquid biopsies, such as serum and urine samples. To achieve this, we propose the use of a two-step liquid-liquid extraction to eliminate interfering compounds and to reduce matrix effects that will become useful for large clinical studies. Here, this extraction procedure was compared with different previously described extraction protocols, all of them combined with dansyl chloride derivatization of polyamines. Moreover, we included several acetylated and diacetylated polyamines and other compounds that were recently described as catabolites of polyamine metabolism [41]. Finally, we applied the developed method to a small trial to validate the method usefulness in a clinical environment.

## 2. Materials and Methods

### 2.1. Solvents, Reagents, and Standards

Acetonitrile (LC-MS grade) was purchased from Merck (Darmstadt, Germany), and methanol and formic acid (both LC-MS grade) were purchased from Sigma-Aldrich (St. Louis, MO, USA). Chloroform, sodium bicarbonate, sodium carbonate, ammonium acetate, trifluoroacetic acid, perchloric acid, sodium hydroxide, acetone, and dansyl chloride were obtained from Sigma-Aldrich, whereas ethyl acetate was obtained from Merck. The water used throughout the study was purified in a Milli-Q system from Millipore (Burlington, MA, USA).

N1-acetylspermidine (N1-AcSPD), N8-acetylspermidine (N8-AcSPD), spermine-d20, spermine, spermidine-d6, spermidine, putrescine-d8, putrescine, N1,N12-diacetylspermine, N1,N8-diacetylspermidine (N1,N8-DiAcSPD), N8-acetylspermidine-d3, N1,N8-diacetylspermidine (N1,N8-DiAcSPD-d6), and agmatine were acquired from Toronto Research Chemicals (North York, ON, Canada). Cadaverine, N-acetylputrescine, N-acetylspermine (N1-AcSPM), lysine, ornithine, arginine, γ-aminobutyric acid, and 1,3-diaminopropane were purchased from Sigma-Aldrich. Lysine (^13^C_6_,^15^N_2_) and arginine (^13^C_6_,^15^N_4_) were supplied by Cambridge Isotope Laboratories (Tewksbury, MA, USA).

Oasis HLB cartridges (1 cc Vac Cartridge, 30 mg sorbent, and 30 µm particle size) were acquired from Waters (Milford, MA, USA).

### 2.2. Preparation of Stock Solutions and Calibration Standards

All stock standard solutions were prepared in MeOH or water depending on the solubility and were stored at −80 °C. The working solutions were prepared from the stock solutions by sampling an aliquot and diluting it with water.

The internal standard working solution (IS) contained a mixture of 0.5 mg/L of spermine-d20, spermidine-d6, putrescine-d8, N8-acetylspermidine-d3, and N1,N8-diacetylspermidine-d6, and 1.25 mmol/L of lysine-lab (^13^C_6_,^15^N_2_) and arginine-lab (^13^C_6_,^15^N_4_) prepared in water.

### 2.3. Serum and Urine Samples

Serum and urine samples were obtained from 40 obese subjects (BMI ≥ 30 kg/m^2^) who were recruited at the Virgen de la Victoria University Hospital (Malaga, Spain) for clinical studies. All participants provided written informed consent, and the study protocol and procedures were approved according to the ethical standards of the Declaration of Helsinki by the Research Ethics Committee of the Virgen de la Victoria University Hospital (Comité de Ética de la Investigación Provincial de Málaga, “Estudio del Papel de las Poliaminas en la Prevención de la Obesidad”, 21st December 2017).

Blood samples were obtained from the antecubital vein after an overnight fast of 12 h and were placed in vacutainer tubes. Excessive agitation of blood specimens was avoided to minimize hemolysis, and the serum was immediately separated from the blood samples by centrifugation for 10 min at 4000 rpm and frozen at −80 °C until analysis. Serum triglycerides, high density lipoprotein (HDL), and fasting glucose levels were analyzed using an Advia Chemistry XPT autoanalyzer (Siemens Healthcare Diagnostics, Erlangen, Germany). Serum glycosylated hemoglobin (A1c) levels were measured using an ADAMS A1c HA-8180T analyzer (ARKRAY, Inc. Amstelveen, The Netherlands). The LDL-cholesterol levels were calculated using the Friedewald formula.

For method development and validation studies in serum and urine, a pool of samples was used as a reference sample across all the study.

### 2.4. Sample Preparation

#### 2.4.1. Protein Precipitation

Aliquots of 100 µL of serum or urine and 5 µL of IS were transferred to a LoBind Eppendorf microcentrifuge tube and vortexed for 30 s. For protein precipitation, we proposed the use of chloroform: methanol (CHCl_3_:MeOH) (2:1, *v*/*v*) and we compared it with the three following methodologies: PCA, MeOH, and ACN. Protein precipitation with PCA was performed by adding 50 µL of 10% PCA. The supernatant was transferred to a new tube and neutralized with 23 µL of 2.5 M NaOH. Protein precipitation with MeOH was carried out by adding 167 µL of MeOH, and the supernatant was transferred to a new tube. For ACN, 400 µL was added, and the supernatant was transferred to a new tube. Finally, protein precipitation with CHCl_3_:MeOH (2:1, *v*/*v*) was carried out by adding 167 µL of MeOH and 333 µL of CHCl_3_, and the upper phase was transferred to a new tube.

#### 2.4.2. Derivatization

The supernatant or upper phase was mixed with 100 µL of carbonate-bicarbonate buffer (0.2 M, pH 9) and 50 µL of dansyl chloride (10 mg/mL in acetone). The mixture was incubated at room temperature for 1 h.

#### 2.4.3. Purification

To purify the derivatized analytes, two methods were tested: LLE with ethyl acetate and SPE. The LLE method was performed by mixing 250 µL of ethyl acetate, and the resulting upper phase was recovered. The sample was acidified with the addition of 2.5 µL of trifluoroacetic acid (TFA), and the extraction step with 250 µL of ethyl acetate was repeated. The combined upper phases were evaporated in a vacuum concentrator at 45 °C and stored at −20 °C until analysis. For the SPE method, the derivatized sample was acidified with 4 µL of TFA and loaded onto an Oasis HLB cartridge (Waters) previously conditioned with 1 mL of ACN and 1 mL of 1% TFA in water. The sample was washed with 1% TFA and dried for 30 min under vacuum. The analytes were eluted twice with 500 µL of ACN, and the eluates were evaporated in a vacuum concentrator at 45 °C and stored at −20 °C until analysis. In both cases, the dried extract was reconstituted with 50 µL of ammonium acetate 0.2 M:ACN (30:70), and 2.5 µL of the supernatant were injected for analysis.

Because of polyamine’s tendency to attach to glass, all the materials used for sample preparation were made of plastic.

### 2.5. Liquid Chromatography and Mass Spectrometry Conditions

LC-MS/MS analysis was performed using an Agilent UHPLC 1290 Infinity II Series coupled to an Agilent QqQ/MS 6490 Series (Agilent Technologies, Sta. Clara, CA, USA). Chromatographic separation was performed using a Kinetex EVO C18 analytical column (2.6 µm; 2.1 mm × 150 mm) (Phenomenex, Torrance, CA, USA). The gradient employed was 0–1.5 min, 0% B; 1.5–7.5 min, 0–40% B; 7.5–9 min, 40% B; 9–15 min, 40–70% B; 15–17.25 min, 70–100% B; 17.25–19 min, 100% B; 19–20 min, 100–0% B; and 20–23 min, 0% B, with the mobile phases being 0.1% formic acid in water (Solvent A) and 0.1% formic acid in ACN (Solvent B). The flow rate was 0.4 mL/min, the injection volume was 2.5 µL and the column temperature was set at 25 °C.

The triple quadrupole operated in the positive electrospray ionization mode (ESI+). The source conditions were set at 15 psi for the nebulizer gas, 200 °C for the gas temperature, 15 L/min for the gas flow, 350 °C for the sheath gas temperature, 11 L/min for the sheath gas flow, 2500 V for the capillary voltage, and 1000 V for the nozzle voltage. Quantitative determination was performed using the multiple reaction monitoring (MRM) mode, and the transitions for each compound are detailed in Table 1.

Simultaneously, to tentatively identify some polyamines, we used an Agilent QTOF/MS high-resolution instrument operating in the ESI+ mode. The source conditions were the same as those used for the QqQ mass spectrometer. Data acquisition was carried out in a full-scan over a mass-range of 50 to 1200 *m/z*, and the fragmentation studies were carried out at 10 V of collision energy.

### 2.6. Method Validation

The developed method using CHCl_3_:MeOH extraction followed by dansyl chloride derivatization and ethyl acetate LLE was validated in serum samples by determining the recovery, accuracy, limits of detection (MDL) and quantification (MQL), linearity, repeatability, reproducibility, and matrix effects. The recovery was determined by comparing the peak areas obtained for the standard after the extraction process with the peak areas obtained for the pure standard. The accuracy was assessed by determining the concentration in serum samples spiked with three different levels according to endogenous levels and linearity range. The MQLs were defined as a signal/noise ratio equal to 10 and the MDLs were defined as a signal/noise ratio equal to 3. Repeatability and reproducibility (expressed as RSD) were evaluated with a pool of non-spiked serum samples prepared and analyzed on the same day (repeatability) and three different days (reproducibility). The matrix effects were evaluated by comparing the response of the spiked serum samples with those of the pure standards. Samples were spiked at a concentration similar to the non-spiked sample for each compound. Pure standards were prepared at the same concentration as the spiked samples. Standards and samples were treated with the same extraction process. Matrix effects were calculated as follows: (((Peak area of spiked sample − peak area of non-spiked sample)/peak area of standard) − 1) * 100.

To assure the quality along the analysis batch, a pool of samples used as quality control were injected regularly to the LC-MS.

## 3. Results and Discussion

### 3.1. Optimization of Chromatography and Mass Spectrometry Conditions

Considering the small size and aliphatic structure of polyamines, dansyl chloride was used as a derivative agent to increase their retention and ionization. The acquisition of dansylated analytes and their stable-isotope-labeled internal standards was performed in positive electrospray ionization mode, and the source conditions were optimized to obtain the maximum response for the [M + H]^+^ ion. The MRM transitions and collision energies were optimized via available standards to obtain the maximum response. Subsequently, the quantitative and qualitative transitions were assessed on selectivity criteria to avoid potential interferences originating from isomeric and isobaric compounds present in the complex serum and urine matrices.

In this sense, the common product ion for all analytes was 170 *m/z*, a characteristic fragment of dansyl chloride. This fragment showed a high response; however, its low selectivity and high background made it only useful as a quantitative transition for LYS, ORN, PUT, and GABA. More specific product ions were selected for each analyte as quantitative transitions. For example, in N-AcPUT, the selected quantitative product was 322 *m/z* from [M-CH_2_=CO]^+^ since the fragment of the derivatizing agent is interfered in serum samples. In the case of N1-AcSPD and N8-AcSPD, distinctive product ions are formed due to SPD’s asymmetrical structure, which allows selectivity between them (654 > 100 *m/z* for N1-AcSPD and 654 > 114 *m/z* for N8-AcSPD). The 360 *m/z* product was used as a quantitative ion in SPM and SPD, the structure and formation mechanisms of which have been described in previous publications [42].

The chromatographic separation of dansylated amino compounds was performed on a Kinetex EVO C18 column packed with superficially porous particles (0.35 µm shell thickness) with a gradient using water and ACN with 0.1% formic acid, based on a method described by Ducros et al. [35]. The gradient used was optimized to improve the separation of analytical standards and to resolve interferences from serum and urine matrices. In the case of GABA, a decrease in the gradient slope was necessary to separate it from the isomeric amino acids, such as α-aminobutyric acid, α-aminoisobutyric acid, and β-aminobutyric acid, which are present in biological fluids. The selected quantitative transition for AGM was 364 > 347 *m/z* from [M + H-H_2_O]^+^, which had lower noise than 364 > 170 *m/z*, but still had an interference next to the analyte. Likewise, the gradient was also modified to resolve the AGM peak from interferences. For this purpose, the matrices were spiked with pure standard since the concentrations of AGM in the serum and urine are very low. In this sense, special attention needs to be paid because the interference peak can easily be confused with AGM if the separation of both peaks is not well resolved.

### 3.2. Sample Extraction Optimization

We tested different sample preparation methods, and they all consisted of three main steps: Protein precipitation, derivatization, and purification. Four different approaches were used for protein precipitation as follows: the proposed LLE with CHCl_3_:MeOH (2:1) and three previously described methods (PCA, ACN, and MeOH). The derivatization of the polyamine species was carried out with dansyl chloride in an alkaline medium. Finally, the purification step was tested with either an LLE or a solid phase extraction (SPE).

#### 3.2.1. Derivatization with Dansyl Chloride and an Alkaline pH

Different derivatization times, temperatures, and pHs were tested, and the most efficient combination was one hour at room temperature and pH 9. A longer derivatization time, higher pH, and/or higher temperature did not make a significant improvement. The pH needs to be alkaline; however, it has been described that at a pH higher than 10, dansyl chloride reacts highly with water or the other hydroxyl groups present in the environment [43]. This means that the majority of dansyl chloride is actually lost by hydrolysis and is not available for the analytes to react with. The optimum pH range for dansyl chloride derivatization is between 8.5 and 9.5. Then, the samples were buffered to a pH of 9 with a carbonate-bicarbonate buffer and were derivatized with dansyl chloride for one hour at room temperature in the dark.

The accurate adjustment of the pH also proved to be important in the final step of the sample preparation. The reconstitution solution needed to be buffered to pH 5.5 to avoid the distortion of the first peak of the chromatogram. We accomplished this with the addition of 0.2 M ammonium acetate buffer.

#### 3.2.2. Purification of the Derivatized Analytes

We tested the different LLE and SPE methods for the purification and concentration of the derivatized analytical standards for the last part of the extraction. The recoveries for each method were evaluated by comparing the peak areas obtained for the standard after the purification process with the peak areas obtained for the pure standard, and the results are shown in Table 2. The LLE with the ethyl acetate method had very good recoveries (over 90%) for all the analytes except for ARG and GABA, and the recoveries for these analytes were very low. Since we were interested in covering the entirety of the polyamine metabolism, we tried acidifying the sample with TFA before the LLE to achieve better recoveries of the more polar analytes. Indeed, LLE with TFA highly improved the recoveries of ARG and GABA but diminished the recoveries of some polyamines. For example, the recovery for SPM decreased from 108 to 52%, and the recovery for N1-AcSPM decreased from 96 to 66%. The SPE showed good recoveries (over 80%) for CAD, GABA, LYS, ORN, PUT, and SPD, but the other analytes did not have better recoveries than those from the LLE. Some of the recoveries were low; for example, ARG and SPM were lower than 40%. Finally, we opted for LLE with ethyl acetate. We decided to extract the analytes twice: The first extraction was performed directly, and the second extraction was acidified with TFA to achieve the best possible recovery for all the analytes.

#### 3.2.3. Protein Precipitation and Matrix Effect

Regarding the first part of the procedure, we compared the extraction with the four approaches mentioned above. PCA, MeOH, and ACN have been applied in previous studies to extract polyamine and related compounds. The sequential extraction of CHCl_3_:MeOH (2:1) based on the methodology of Folch et al. [44] is proposed to improve the matrix effect due to its lipid removal effect.

In terms of area, the highest results were achieved by CHCl_3_:MeOH and PCA, and MeOH and ACN showed the lowest areas with the exception of MeOH in LYS, ORN, 1,3-DAP, and PUT, which showed areas similar to that of CHCl_3_:MeOH (data shown in Figure S1 in the Supplementary Material). In relation to the extraction efficiencies, all extractions showed similar capacities, and the observed differences in the area were due to matrix effects. Although protein precipitation with PCA is a widely used method for the analysis of polyamines [23,25,35,36], its repeatability in serum extraction was poor, becoming especially critical for compounds for which there are no labeled internal standards. For instance, the relative standard deviation (RSD) for the majority of the analytes for PCA extraction is over 40%, with the highest being 74.1% for N1-AcSPM, 69.7% for AGM, and 62.3% for SPM (data shown in Figure S1); whereas for CHCl_3_:MeOH extraction, the RSD was lower than 20% for all the compounds, with the exception of AGM (28.6%) and N1,N12-DiAcSPM (24.8%).

**Figure 1:**
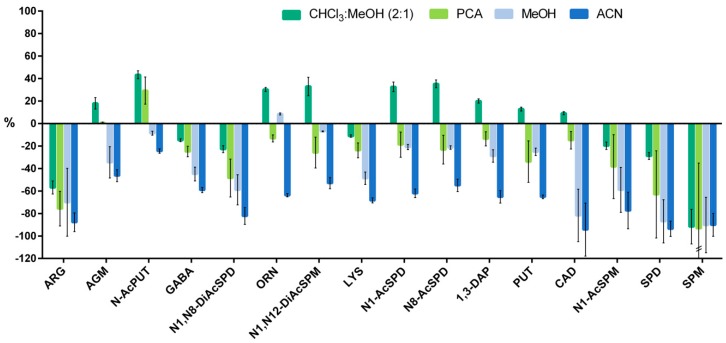
Comparison of matrix effects (%) in serum samples with the four approaches assessed to extract polyamines and related compounds (*n* = 3). The *Y* axis indicates the matrix effects in percentage. Positive values indicate ion enhancement and negative values indicate ion suppression. Metabolites in the *X* axis are presented in the order of elution.

The matrix effects were determined for the four methodologies. The results from the serum samples are shown in Figure 1. ACN and MeOH presented the highest matrix effects for most of the analytes. The four extraction solvents showed similar and very high matrix effects in the cases of ARG and SPM, which strengthens the need for the use of labeled internal standards. The MeOH and CHCl_3_:MeOH methods showed similar areas and matrix effects in the most polar analytes, but interestingly, the nonpolar analytes had higher matrix effects with MeOH extraction due to the coelution of lipid compounds. With the addition of CHCl_3_ to the extraction mixture, we were able to eliminate the lipids present in the serum and to lower the matrix effects of the less polar analytes. These improvements were not observed in the first compounds to elute, given the greater polarity of these dansylated polyamines. Figure 1 shows that a signal enhancement was present for some of the analytes with the PCA and CHCl_3_:MeOH methods instead of signal suppression. This phenomenon has already been described for polyamines in biological matrices by Häkkinen et al. [20]. The extraction with PCA showed low matrix effects similar to those of the CHCl_3_:MeOH method. Its similar behavior to that of the CHCl_3_:MeOH method may be due to PCA’s low extraction capacity and the deleterious effects of the lipid components [45].

The four extractions that were quantified using internal standards showed similar concentrations. Despite the similarity, the best performance was shown by the CHCl_3_:MeOH method due to its higher sensitivity, sample clean-up ability, and high repeatability. For instance, the higher sensitivity of the CHCl_3_:MeOH extraction is noteworthy in analytes, such as N1-AcSPM, CAD, or N1,N8-DiAcSPD, because of its low concentration in serum samples.

Focusing on the behavior of the analytes with CHCl_3_:MeOH extraction, there are a few similarities that will be of interest when choosing the best internal standard for each of them. For instance, N-AcPUT, ORN, N1,N12-DiAcSPM, N1-AcSPD, and N8-AcSPD have comparable signal enhancement. Additionally, PUT’s behavior is very similar to the behavior of CAD and 1,3-DAP, and GABA and N1,N8-DiAcSPD show similar signal suppression. In addition, contrary to what one might expect, N1-AcSPM shows matrix effects more similar to SPD than to SPM. Since N1-AcSPM is a product of SPM acetylation, their structures are similar, and they would be expected to behave the same way. However, acetylation gives N1-AcSPM more polarity upon derivatization, and makes it elute close to SPD and almost two minutes earlier than SPM in a different region of the chromatogram. The same pattern occurs with the other polyamines and their respective acetylated forms, and the differences between them are more evident the smaller the polyamine is.

### 3.3. Identification

A semitargeted approach was used to search for other acetylated polyamines and related metabolites in human serum and urine samples, such as N-acetylcadaverine (AcCAD), N-acetyldiaminopropane (AcDAP), and N1-acetylisoputreanine (N1-AcIsoPUTR). The instrument used was an Agilent 6550 QTOF, and the chromatographic conditions were the same as those used in the targeted approach. The identification was based on exact mass, chromatographic behavior, fragmentation pattern, and published data. To tentatively identify AcDAP and AcCAD, the expected dansylated precursor ions were observed with an error <5 ppm, and the product ion spectra showed the characteristic daughter ions described by Gottfried Feistner [42]. The 308 and 336 *m/z* ions are obtained from [M-CH_2_=CO]^+^, and the 58 and 86 *m/z* ions are obtained from the subsequent loss of the amino dansyl group (DnsNH_2_) for AcDAP and AcCAD, respectively. In addition, the chromatographic behavior of AcDAP (6.98 min) and AcCAD (7.54 min) with respect to 1,3-DAP (12.84 min) and CAD (13.22 min) was consistent with the behaviors of AcPUT (7.93 min) and PUT (13.81 min), for which we had commercial standards.

**Figure 2:**
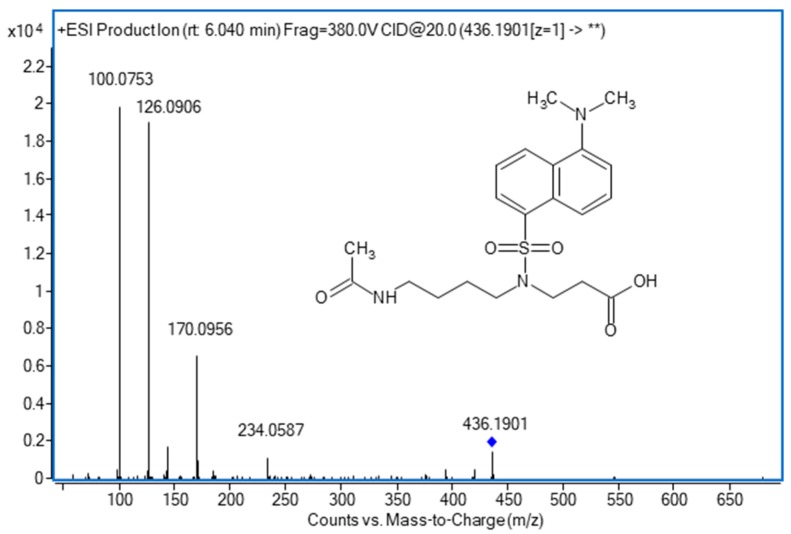
N1-acetylisoputreanine (N1-AcIsoPUTR) product ion spectrum at 30 V of collision energy.

The other tentatively identified compound was N1-AcIsoPUTR, which is an end-product of polyamine metabolism first described in urine by Fitzgerald et al. [41]. The expected dansylated precursor ion was observed with an error lower than 5 ppm, and the main fragment from the loss of dansylated GABA at 100.0753 *m/z* [M + H-Dns-C_4_H_9_NO_2_(GABA)] was also detected with an error <5 ppm (Figure 2). The product ion of 126.0906 *m/z* has also been described by Fitzgerald et al. [41].

These three tentatively identified metabolites were included in the final methodology and were semiquantified in urine and serum samples.

### 3.4. Method Validation

The method validation parameters were determined as explained in Section 2.6 and are shown in Table 3. The MDLs were between 0.004 and 0.4 nM for the polyamines (AGM, PUT, SPD, SPM, 1,3-DAP, CAD, and acetylated derivatives) and between 0.8 and 22.0 nM for the amino acids (ARG, LYS, ORN, and GABA). The MQLs ranged from 0.01 to 1.40 nM for the polyamines and from 2.8 to 74.0 nM for the amino acids. With respect to polyamines, these values are lower than what the majority of published methods have achieved. For instance, Wong et al. [24] used benzoyl chloride derivatization in serum samples and reported an MDL of 2 nM. Ducros et al. [35] used dansyl chloride derivatization in red blood cells and described an MDL of 0.1 nM for putrescine, 0.75 nM for spermidine, and 0.5 nM for spermine. Liu et al. [4] obtained an MQL of 0.1 to 0.6 ng/mL in plasma and urine with ion pairing chromatography, and Xiong et al. [28] used FMOC derivatization in plasma and reported an MQL of 0.2 to 2 ng/mL. The repeatability and reproducibility were below 20% for most of the compounds, except for 1,3-DAP reproducibility, which was 21.2%. The recoveries of all polyamine and precursors were above 82% in the serum matrix. Matrix effects were already discussed in Section 3.2 and are shown in Figure 1.

The application of the method in the urine matrix was also assessed and showed a good performance (the results are shown in Supplementary Table S1). In all cases, the matrix effects in the urine samples were below 20% and the recoveries were above 80%. It should be mentioned that for urine, only 1 µL was injected due to the higher concentration of the compounds. The values of repeatability and reproducibility in all cases were below 20%.

In both serum and urine samples, we observed that the use of internal standards is essential for the analysis of polyamines. In most of the previously published works, when an internal standard is used, it is usually 1,6-diaminohexane. In this sense, we found that it can be helpful to use more than one internal standard and that it is preferable to use stable isotope-labeled standards for this purpose. Publications, such as that by Samejima et al. [39], have used N1-AcSPD- and N1,12-DiAcSPM-labeled internal standards; Stevens et al. [18] used ORN-, PUT-, SPD-, and SPM-labeled internal standards; Häkkinen et al. [20] used 14 labeled polyamines and acetylated polyamines; and Tomita et al. [21] used numerous labeled polyamines and amino acids. In the current method, we used SPM, SPD, PUT, N8-AcSPD, N1,N8-DiAcSPD, LYS, and ARG stable isotope-labeled standards, and its use proved to be essential for the repeatability of the method.

For the analytes that had no corresponding stable isotope-labeled compound, the assignment of the best internal standard was performed considering the similarity in structure and retention time. Evaluating the behavior of the analytes with respect to the matrix effects also proved to be useful, as previously discussed. Overall, the goal is to select the internal standard that will behave more similarly to the analyte and will provide a better compensation for the matrix effects. The internal standard assigned to each analyte can be seen in Table 1.

### 3.5. Method Application

**Figure 3:**
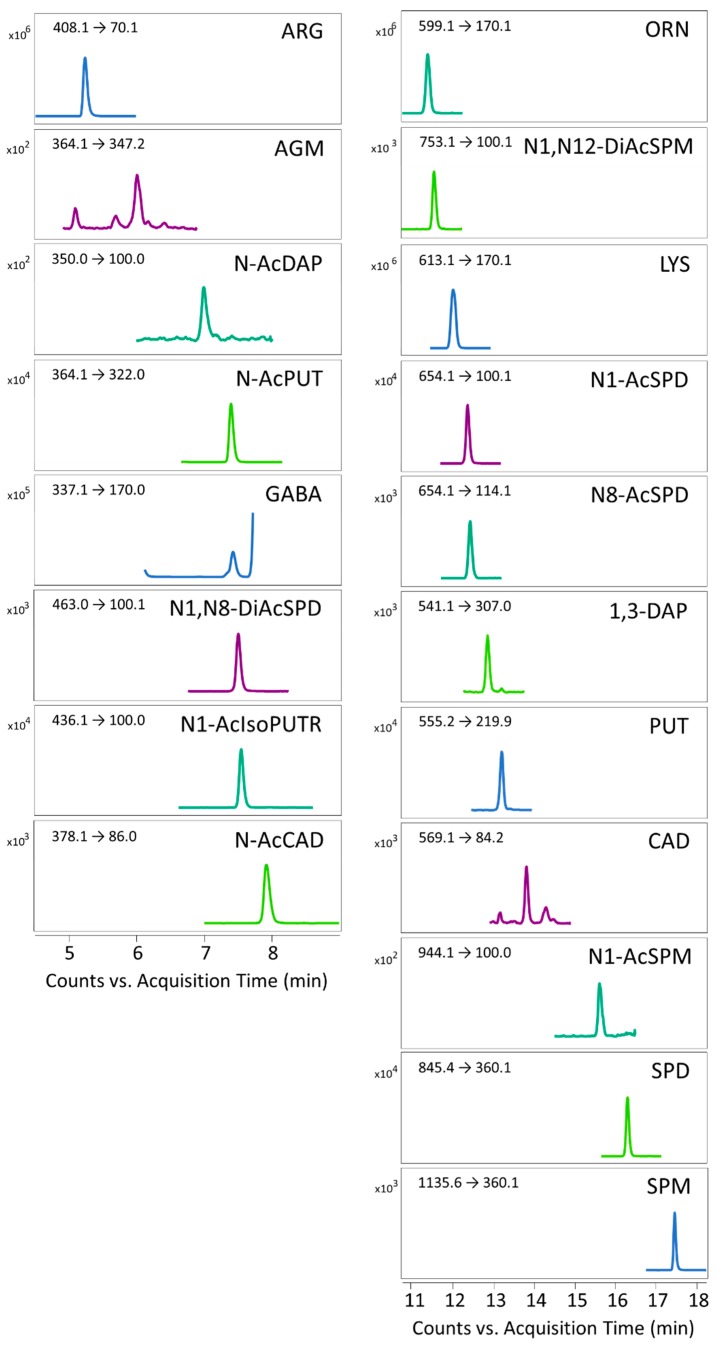
Chromatogram of a serum sample with the quantitative transition used for each analyte.

The developed method was applied to investigate the polyamine metabolism in human serum and urine samples on a gender-related trial, consisting on 40 obese patients, of whom, 17 were men and 23 were women. The clinical characteristics of these subjects are shown in Supplementary Table S2. The concentration values of polyamines, precursors, and catabolites measured by the current method are shown in Table 4 and Supplementary Figure S2 and are in accordance with those reported in the literature in serum [24,26,27] and urine [14,26,46]. In Figure 3, an MRM chromatogram of a serum sample for the 19 determined compounds is shown.

Hence, the serum levels of polyamines and acetylated products are much lower than those for related amino acids, such as ARG, ORN, and LYS (nM range vs. µM for amino acids). Among the polyamines analyzed in serum by our platform, SPD was the most predominant (152.5 ± 63.7 nM), although significant levels of other polyamines, such as PUT (77.7 ± 28.3 nM), SPM (25.4 ± 10.0 nM), and the SPD acetylated forms N1-AcSPD (76.8 ± 27.4 nM) and N8-AcSPD (35.4 ± 8.0 nM) were also found in circulation. To our knowledge, this is the first time that N1,N8-DiAcSPD, N1,N12-DiAcSPM, and N1-AcIsoPUTR were detected and quantified in human serum. In urine, however, the levels of N-AcPUT (15.9 ± 6.9 nmol/mg creatinine) were similar to those of related amino acids, such as ARG (25.9 ± 20.3 nmol/mg creatinine) and ORN (18.2 ± 29.0 nmol/mg creatinine). Overall, the urine polyamine metabolome mainly consists of acetylated polyamines, such as N-AcPUT, N-AcCAD, N1-AcSPD, and N8-AcSPD, with only significant levels of SPM in men. The N-AcCAD, N-AcDAP, and N1-AcIsoPUTR levels were semiquantified with the current method in urine and serum samples with CAD, DAP, and GABA calibration curves, respectively.

The sex difference of each metabolite in the study subjects was examined by Student’s *t*-test. The levels of SPM in both serum and urine were significantly higher in men compared to those in women (*p* < 0.01). In contrast, the levels of 1,3-DAP, PUT, and CAD in urine were significantly lower in men than those in women (*p* < 0.01). Moreover, the levels of N1-AcSPM in serum and the levels of N-AcCAD, GABA, and N8-AcSPD in urine were also significantly different between men and women (*p* < 0.05). Previous articles have described sex differences in the levels of some polyamines in certain biofluids. For example, marked sex differences for N-acetylated polyamines in human urine were identified by Paik et al. [13]. Ibrahim et al. [47] reported that the SPD and SPM serum levels differed significantly according to sex in atherosclerosis patients. In contrast, Magnes et al. [27] assessed sex differences in the human serum samples of obese patients and found no significant differences in polyamine concentration between males and females.

## 4. Conclusions

Here, we have described a robust and high-throughput LC-MS/MS method for the simultaneous determination of 19 metabolites related to polyamine metabolism at levels ranging from trace amounts to high concentrations. The sample preparation was optimized to eliminate interfering compounds and to reduce matrix effects; in addition, it combines dansyl chloride derivatization with two solvent extraction and partitioning steps. The first step (prior to derivatization) removes lipids based on Folch’s extraction, and in the second step (after derivatization), ethyl acetate isolates the derivatized polyamines from more polar interfering compounds. The proposed method was applied to human serum and urine samples, and all the analytes were successfully quantified. Three acetylated polyamines were tentatively identified in urine samples and were semiquantified in both serum and urine samples. The results demonstrate that the developed method can be used to analyze biological samples for the study of polyamine metabolism and its association with human diseases.

## Figures and Tables

**Table 1:** Selected multiple reaction monitoring (MRM), collision energy (CE), retention time, and internal standard (IS) used for each analyte.

Analyte	Retention Time (min)	Precursor Ion (*m/z*)	Product Ion (*m/z*)	CE	IS
ARG	5.2	408.1	70.1	40	ARG-lab
			170.3	32	
AGM	5.7	364.1	347.2	20	ARG-lab
			170.1	28	
			155.2	52	
N-AcDAP	6.9	350.0	100.0	20	N8-AcSPD-lab
			170.0	20	
			308.0	20	
N-AcPUT	7.4	364.1	322.0	15	N8-AcSPD-lab
			170.1	28	
			234.0	15	
GABA	7.4	337.1	170.0	20	ARG-lab
			296.0	8	
			157.0	36	
N1,N8-DiAcSPD	7.5	463.0	100.1	28	N1,N8-DiAcSPD-lab
			114.1	28	
			170.2	40	
N1-AcIsoPUTR	7.5	436.1	100.0	10	ARG-lab
			126.2	20	
			170.0	30	
N-AcCAD	7.9	378.1	86.0	20	N8-AcSPD-lab
			155.0	50	
			335.9	20	
ORN	11.5	599.1	170.1	48	LYS-lab
			303.1	24	
N1,N12-DiAcSPM	11.6	753.1	100.1	32	N1,N8-DiAcSPD-lab
			502.3	40	
			170.0	60	
LYS	12.1	613.1	170.1	52	LYS-lab
			317.1	24	
N1-AcSPD	12.4	654.1	100.1	20	N8-AcSPD-lab
			305.1	20	
N8-AcSPD	12.4	654.1	114.1	20	N8-AcSPD-lab
			541.1	20	
1,3-DAP	12.9	541.1	307.0	24	SPD-lab
			170.2	36	
			220.0	24	
PUT	13.3	555.2	219.9	28	PUT-lab
			170.1	44	
CAD	13.8	569.1	84.2	36	PUT-lab
			170.2	44	
			186.0	36	
N1-AcSPM	15.7	944.1	100.0	44	SPM-lab
			693.3	52	
			360.2	56	
SPD	16.3	845.4	360.1	40	SPD-lab
			170.1	60	
SPM	17.5	1135.6	360.1	56	SPM-lab
			170.1	60	
ARG-lab	5.2	418.1	170.3	32	N/A
LYS-lab	12.1	621.1	170.1	52	N/A
N8-AcSPD-lab	12.4	657.0	117.2	40	N/A
			170.0	60	
			362.2	40	
N1,N8-DiAcSPD-lab	7.5	469.0	103.1	28	N/A
			117.1	28	
			170.1	40	
PUT-lab	13.3	563.2	219.9	28	N/A
			170.1	44	
SPD-lab	16.3	851.1	366.2	40	N/A
			170.2	60	
SPM-lab	17.5	1155.6	373.1	56	N/A
			170.1	60	

N/A: not applicable.

**Table 2:** Recoveries in the percentage of the derivatized analytes for the three purification methods tested. (*n* = 3) * EA: ethyl acetate.

Analyte	LLE:EA *	LLE:EA + TFA	SPE
ARG	4 ± 3	75 ± 11	20 ± 8
AGM	108 ± 6	97 ± 7	50 ± 5
N-AcPUT	97 ± 6	93 ± 13	46 ± 6
GABA	1 ± 2	153 ± 37	115 ± 10
ORN	120 ± 15	92 ± 8	92 ± 7
LYS	171 ± 62	128 ± 10	124 ± 8
N1-AcSPD	99 ± 11	81 ± 7	75 ± 5
N8-AcSPD	90 ± 8	73 ± 5	66 ± 9
1,3-DAP	94 ± 7	64 ± 8	62 ± 7
PUT	108 ± 9	83 ± 11	83 ± 4
CAD	105 ± 9	83 ± 9	82 ± 5
N1-AcSPM	96 ± 11	66 ± 6	57 ± 7
SPD	161 ± 53	100 ± 7	98 ± 6
SPM	108 ± 7	52 ± 5	37 ± 3

**Table 3:** Quality parameters for the analytes in serum using the proposed method.

Analyte	Recovery (%, *n* = 5)	Accuracy (% *n* = 3)	MDL ^1^ (nM)	MQL ^2^ (nM)	Repeatability (%RSD *n* = 5)	Reproducibility (%RSD *n* = 3)	Linear Range (nM)	R^2^
ARG	82 ± 2.4	114.7 ± 2.2	22.14	73.81	4.3	1.0	75937.4–759373.5	0.9998
AGM	103 ± 7.1	84.3 ± 6.5	0.04	0.14	6.7	15.6	7.7–15.4	0.9961
N-AcDAP	N/A	N/A	N/A	N/A	9.8	12.9	N/A	N/A
N-AcPUT	98 ± 6.7	115.2 ± 10.1	0.10	0.35	8.8	1.9	18.0–180.0	0.9983
GABA	110 ± 3.7	95.7 ± 14.9	1.08	3.60	5.7	3.7	290.9–581.8	0.9944
N1,N8-DiAcSPD	107 ± 8.3	101.3 ± 4.3	0.01	0.03	9.1	1.3	0.9–43.6	0.9999
N1-AcIsoPUTR	N/A	N/A	N/A	N/A	18.3	12.8	N/A	N/A
N-AcCAD	N/A	N/A	N/A	N/A	3.3	1.2	N/A	N/A
ORN	92 ± 4.1	109 ± 3.8	0.84	2.81	2.5	1.3	94887.9–948879.1	0.9936
N1,N12-DiAcSPM	111 ± 4.8	101.2 ± 7.2	0.004	0.01	4.3	1.3	0.6–5.6	0.9962
LYS	121 ± 3.4	107.9 ± 1.6	1.99	6.66	2.4	5.9	109446.6–1094466.1	0.9959
N1-AcSPD	95 ± 4.5	101.4 ± 14.3	0.02	0.08	3.5	3.3	115.3–5764.8	0.9995
N8-AcSPD	88 ± 5.1	98.3 ± 3.5	0.01	0.03	4.2	6.1	115.3–230.6	0.9999
1,3-DAP	96 ± 11.2	119.7 ± 5	0.41	1.38	3.7	21.2	20.4–102.0	0.9855
PUT	93 ± 8.1	99.5 ± 3.8	0.15	0.51	4.2	3.6	113.4–567.2	0.9990
CAD	93 ± 4.8	101.2 ± 3.9	0.03	0.09	3.6	7.8	9.8–19.6	0.9998
N1-AcSPM	87 ± 7.8	104.4 ± 6.6	0.02	0.08	5.6	8.8	2.8–14.1	0.9999
SPD	98 ± 5.8	115.7 ± 3.9	0.01	0.05	7.1	12.1	68.8–3442.3	0.9999
SPM	100 ± 5.6	118.4 ± 6.5	0.04	0.14	2.1	0.9	14.8–296.5	0.9992

^1^ Method detection limit (MDL). ^2^ Method quantification limit (MQL). Not applicable (N/A).

**Table 4:** Amounts of polyamines, polyamine precursors, and catabolites in serum and urine from 40 obese patients.

Analyte (Mean ± SD)	Serum (nM)				Urine (nmol/mg Creatinine)			
	Obese Male Patients	Obese Female Patients	FC ^1^	*p*-Value	Obese Male Patients	Obese Female Patients	FC	*p*-Value
ARG	117.3 × 103 ± 27.6 × 10^3^	114.9 × 103 ± 19.9 × 10^3^	-	>0.05	28.0 ± 25.1	24.4 ± 15.6	-	>0.05
AGM	0.1 ± 0.1	0.1 ± 0.1	-	>0.05	0.2 × 10−3 ± 0.3 × 10−3	0.8 × 10−3 ± 3.4 × 10−3	-	>0.05
N-AcDAP	1.7 ± 0.6	1.8 ± 0.6	-	>0.05	0.6 ± 0.4	0.8 ± 0.4	-	>0.05
N-AcPUT	22.5 ± 8.4	23.6 ± 8.0	-	>0.05	14.7 ± 6.5	16.8 ± 7.0	-	>0.05
GABA	214.9 ± 60.4	192.5 ± 50.5	-	>0.05	0.5 ± 0.2	0.9 ± 0.4	1.54	≤0.05 *
N1,N8-DiAcSPD	0.8 ± 0.6	0.8 ± 0.5	-	>0.05	0.9 ± 0.4	1.0 ± 0.5	-	>0.05
N1-AcIsoPUTR	10.1 ± 6.3	8.0 ± 5.2	-	>0.05	0.4 ± 0.2	0.4 ± 0.3	-	>0.05
N-AcCAD	9.3 ± 12.3	13.6 ± 19.9	-	>0.05	5.2 ± 7.3	11.0 ± 14.5	2.08	≤0.05 *
ORN	151.7 × 10^3^ ± 29.7 × 10^3^	155.7 × 10^3^ ± 30.0 × 10^3^	-	>0.05	22.8 ± 43.4	14.8 ± 6.9	-	>0.05
N1,N12-DiAcSPM	0.7 ± 0.4	0.7 ± 0.4	-	>0.05	0.2 ± 0.2	0.2 ± 0.1	-	>0.05
LYS	308.7 × 10^3^ ± 44.9 × 10^3^	323.9 × 10^3^ ± 41.6 × 10^3^	-	>0.05	0.3 × 10^3^ ± 0.4 × 10^3^	0.1 × 10^3^ ± 0.1 × 10^3^	-	>0.05
N1-AcSPD	81.3 ± 22.8	73.5 ± 29.9	-	>0.05	4.5 ± 2.2	5.4 ± 2.3	-	>0.05
N8-AcSPD	37.0 ± 8.3	34.2 ± 7.6	-	>0.05	4.7 ± 1.4	5.8 ± 1.3	1.24	≤0.05 *
1,3-DAP	8.4 ± 2.5	9.1 ± 4.0	-	>0.05	0.1 ± 0.1	0.2 ± 0.1	1.91	≤0.01 **
PUT	76.5 ± 28.5	78.6 ± 28.1	-	>0.05	0.7 ± 0.6	1.4 ± 1.0	2.51	≤0.01 **
CAD	1.1 ± 0.8	1.3 ± 1.0	-	>0.05	0.1 ± 0.2	0.6 ± 0.8	7.32	≤0.01 **
N1-AcSPM	1.8 ± 0.7	1.4 ± 0.5	1.33	≤0.05 *	4.7 × 10^−3^ ± 4.9 × 10^−3^	8.8 × 10^−3^ ± 11.1 × 10^−3^	-	>0.05
SPD	176.8 ± 79.3	134.5 ± 40.6	-	>0.05	0.3 ± 0.3	0.2 ± 0.2	-	>0.05
SPM	31.6 ± 9.8	20.9 ± 7.4	1.52	≤0.01 **	3.0 ± 3.7	0.1 ± 0.1	14.38	≤0.01 **

^1^ Fold change (FC). FC is only specified for the statistically significant analytes (*p* ≤ 0.05). Otherwise it has been indicated as “-”. * Significant at *p* ≤ 0.05. ** Significant at *p* ≤ 0.01.

## Supplementary Materials

The following are available online at https://www.mdpi.com/2218-273X/9/12/779/s1, Table S1: Intraday (repeatability) and interday (reproducibility) precision for the urine matrix., Table S2: Clinical characteristics of the participants included in the analysis., Figure S1: Comparison of the response and its variability for each extraction method assessed., Figure S2: Scatter dot plot representations of the polyamine levels in the male and female groups. In all the cases represented, there were significant differences in the levels between the study groups (*p* = 0.05).
