# Supplementary material for: Gender-Related Differences on Polyamine Metabolome in Liquid Biopsies by a Simple and Sensitive Two-Step Liquid-Liquid Extraction and LC-MS/MS
Source: Biomolecules. 2019 Nov 26;9(12):779. doi: 10.3390/biom9120779 (PMC6995533; doi:10.3390/biom9120779)

Supplementary data

Table S1. Intraday (repeatability) and interday (reproducibility) precision for the urine matrix.

Analyte	Repeatability (%RSD* n=5)	Reproducibility (%RSD n=3)
ARG	4.2	6.5
AGM	3.5	8.8
N-AcDAP	6.2	11.2
N-AcPUT	9.0	9.1
GABA	4.8	19.2
N1,N8-DiAcSPD	5.6	7.1
N1-AcIsoPUTR	15.5	19.2
N-AcCAD	5.5	4.5
ORN	5.6	16.2
N1,N12-DiAcSPM	4.1	14.2
LYS	3.7	6.1
N1-AcSPD	5.8	17.4
N8-AcSPD	4.9	15.6
1,3-DAP	8.0	16.7
PUT	4.7	6.4
CAD	8.8	10.7
N1-AcSPM	11.2	16.2
SPD	3.2	5.2
SPM	6.9	5.7

*Relative standard deviation (RSD)

Table S2. Clinical characteristics of the participants included in the analysis.

	Average (SD*)
Age, years	65.5 (4.1)
BMI, mg/kg^2	32.6 (3.2)
Systolic blood pressure, mm Hg	137.7 (14.7)
Diastolic blood pressure, mm Hg	75.6 (8.6)
Glucose, mg/dL	99.1 (7.4)
HbA1c, %	5.7 (0.3)
Total cholesterol, mg/dL	200.4 (32.2)
HDL-cholesterol, mg/dL	50.2 (11.2)
LDL-cholesterol, mg/dL	120.6 (28.2)
Triglycerides, mg/dL	148.0 (29.8)

* Standard deviation (SD).

Figure S1. Comparison of the response and its variability for each extraction method assessed in serum samples.

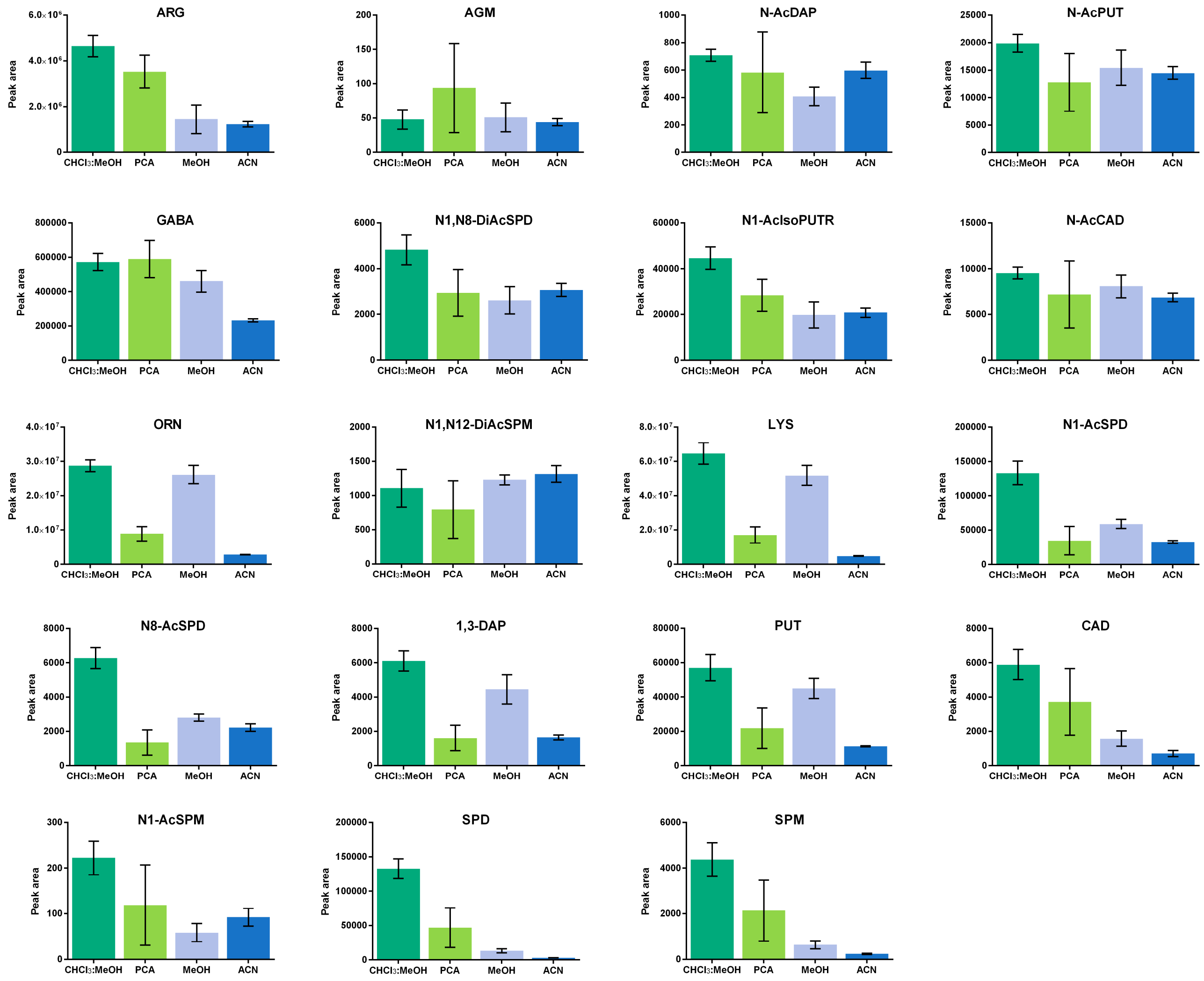

Figure S2. Scatter dot plot representations of the polyamine levels in the male and female groups. In all the cases represented, there were significant differences in the levels between the study groups (p = 0.05).

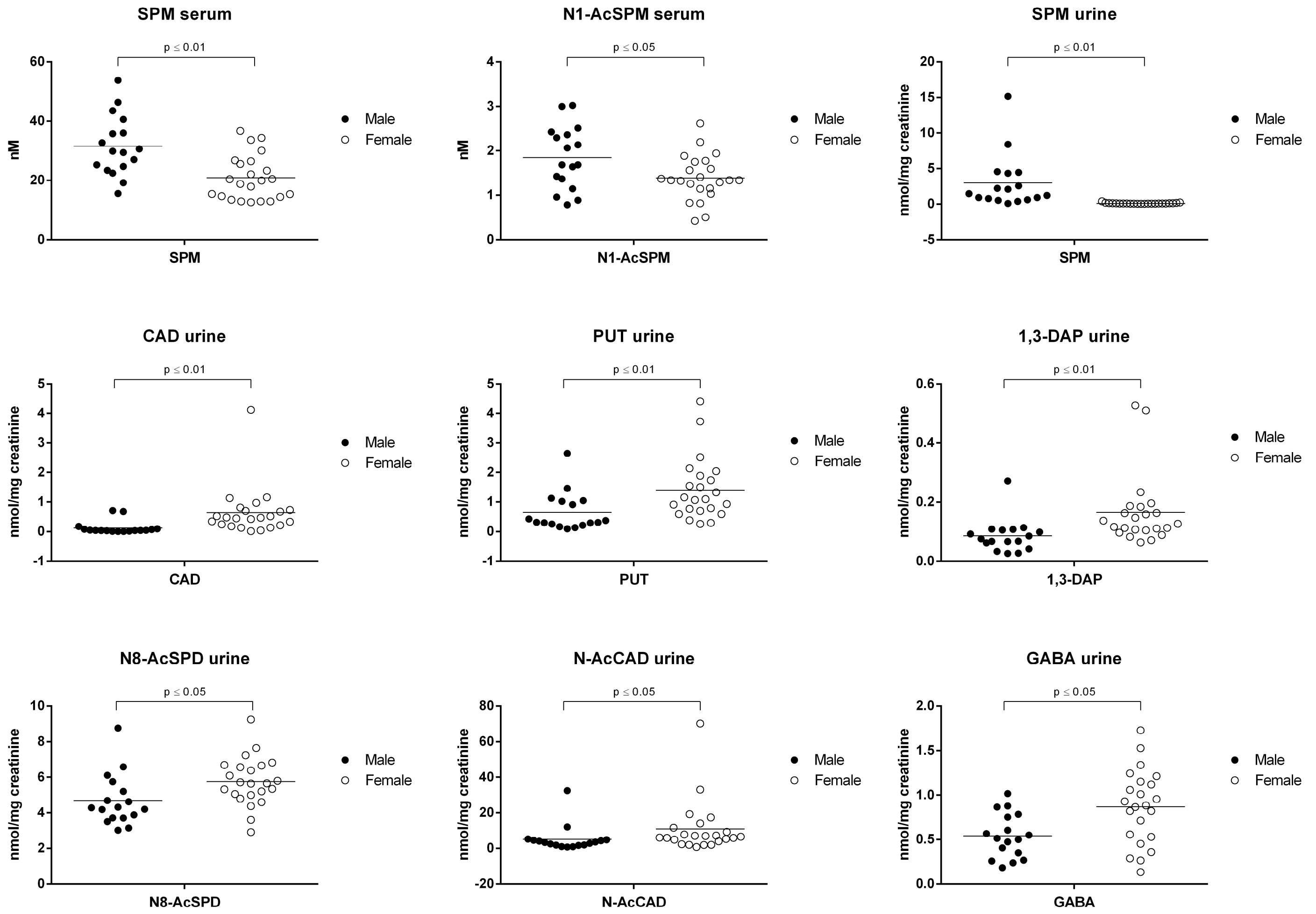

Supplement: Supplementary file 1 [file biomolecules-09-00779-s001.pdf]
